# Supplementary material for: Leveraging machine learning to evaluate factors influencing vitamin D insufficiency in SLE patients: A case study from southern Bangladesh
Source: PLOS Glob Public Health. 2023 Oct 31;3(10):e0002475. doi: 10.1371/journal.pgph.0002475 (PMC10617712; doi:10.1371/journal.pgph.0002475)
Supplement: S1 File — (DOCX) [file pgph.0002475.s001.docx]

STROBE Statement—checklist of items that should be included in reports of observational studies

|  | Item No. | Recommendation | Page  No. | Relevant text from manuscript |
| --- | --- | --- | --- | --- |
| **Title and abstract** | 1 | (*a*) Indicate the study’s design with a commonly used term in the title or the abstract | 1 | Vitamin D; SLE; Machine learning; |
|  |  | (*b*) Provide in the abstract an informative and balanced summary of what was done and what was found | 2 | SLE patients have a high prevalence of vitamin D insufficiency |
| Introduction | | | |  |
| Background/rationale | 2 | Explain the scientific background and rationale for the investigation being reported | 3-5 | Relationship between low vitamin D levels and SLEDAI scores in Systemic Lupus Erythematosus (SLE) patients in Bangladesh. |
| Objectives | 3 | State specific objectives, including any prespecified hypotheses | 6 | identify the variables that significantly contribute to low vitamin D levels among SLE patients. |
| Methods | | | |  |
| Study design | 4 | Present key elements of study design early in the paper | 6 | Case control study |
| Setting | 5 | Describe the setting, locations, and relevant dates, including periods of recruitment, exposure, follow-up, and data collection | 6 | Chattogram Medical College Hospital (CMCH) |
| Participants | 6 | (*a*) *Cohort study*—Give the eligibility criteria, and the sources and methods of selection of participants. Describe methods of follow-up  *Case-control study*—Give the eligibility criteria, and the sources and methods of case ascertainment and control selection. Give the rationale for the choice of cases and controls  *Cross-sectional study*—Give the eligibility criteria, and the sources and methods of selection of participants | 7 | A patient group of 50 patients. criteria for SLE classification and all patients who consented to participate in the study. |
|  |  | (*b*) *Cohort study*—For matched studies, give matching criteria and number of exposed and unexposed  *Case-control study*—For matched studies, give matching criteria and the number of controls per case |  |  |
| Variables | 7 | Clearly define all outcomes, exposures, predictors, potential confounders, and effect modifiers. Give diagnostic criteria, if applicable | 7 | The study performed CBC, ESR, CRP, ANA, anti-dsDNA, vitamin D level or 25(OH)D, 24-hour urinary total protein, and renal Lupus nephritis patient |
| Data sources/ measurement | 8* | For each variable of interest, give sources of data and details of methods of assessment (measurement). Describe comparability of assessment methods if there is more than one group | 8 | Electrochemiluminescence immunoassay method in the serum of SLE patients. |
| Bias | 9 | Describe any efforts to address potential sources of bias | 7 | Patients with certain pre-existing conditions such as End-Stage Renal Disease, Diabetes Mellitus, severe sepsis, SLE with overlap, osteoporosis, osteomalacia, first-degree relatives of SLE or any connective tissue disease, and individuals with psychiatric conditions were excluded from the study as these conditions could potentially affect the results and skew the findings. |
| Study size | 10 | Explain how the study size was arrived at | 7 | The small sample size is considered to be a result of time constraints and limited funding resources, which made it difficult to conduct a larger study. |

Continued on next page

| Quantitative variables | 11 | Explain how quantitative variables were handled in the analyses. If applicable, describe which groupings were chosen and why | 7 | The study collected qualitative data from the patients through face-to-face interviews in the Medicine, Rheumatology, Nephrology, and Dermatology wards of the CMC hospital. A pre-tested questionnaire was used for this study, and all demographic data, such as age, gender, educational status, geographic area, and monthly family income were recorded. Clinical data, including drugs, duration of sun exposure (hours/day), and SLEDAI were also recorded. Laboratory testing data of the patients were also collected from the sample testing laboratory of the hospital. The following link provides free access to the data. |
| --- | --- | --- | --- | --- |
| Statistical methods | 12 | (*a*) Describe all statistical methods, including those used to control for confounding | 8 | *Machine Learning (ML) Approach* |
|  |  | (*b*) Describe any methods used to examine subgroups and interactions |  | *Machine Learning (ML) Approach* |
|  |  | (*c*) Explain how missing data were addressed | N0 |  |
|  |  | (*d*) *Cohort study*—If applicable, explain how loss to follow-up was addressed  *Case-control study*—If applicable, explain how matching of cases and controls was addressed  *Cross-sectional study*—If applicable, describe analytical methods taking account of sampling strategy |  |  |
|  |  | (*e*) Describe any sensitivity analyses | 8-9 | Machine learnining , Heat map |
| Results | | | | |
| Participants | 13* | (a) Report numbers of individuals at each stage of study—eg numbers potentially eligible, examined for eligibility, confirmed eligible, included in the study, completing follow-up, and analysed | 13-15 |  |
|  |  | (b) Give reasons for non-participation at each stage |  |  |
|  |  | (c) Consider use of a flow diagram | 13-15 |  |
| Descriptive data | 14* | (a) Give characteristics of study participants (eg demographic, clinical, social) and information on exposures and potential confounders | 14 |  |
|  |  | (b) Indicate number of participants with missing data for each variable of interest |  |  |
|  |  | (c) *Cohort study*—Summarise follow-up time (eg, average and total amount) |  |  |
| Outcome data | 15* | *Cohort study*—Report numbers of outcome events or summary measures over time |  |  |
|  |  | *Case-control study—*Report numbers in each exposure category, or summary measures of exposure |  |  |
|  |  | *Cross-sectional study—*Report numbers of outcome events or summary measures | *14-16* | As demonstrated through the previously mentioned descriptive statistics, t-test, and correlation results, variables such as Hb, SLEDAI, and sun exposure display significant relationships with vitamin D levels, while other variables like age, sex, BMI, sun protection measures, and CRP exhibit weak or negligible correlation. |
| Main results | 16 | (*a*) Give unadjusted estimates and, if applicable, confounder-adjusted estimates and their precision (eg, 95% confidence interval). Make clear which confounders were adjusted for and why they were included | 17 | Displays the most influential features in descending order based on the magnitude of SHAP values across the test dataset. The blue and red dots situated to the right of zero represent a positive influence on vitamin D levels, while dots to the left signify a negative impact on the predictions. Local explanations of the predictions are provided in Figure 8, illustrating the contributions of individual features. |
|  |  | (*b*) Report category boundaries when continuous variables were categorized |  |  |
|  |  | (*c*) If relevant, consider translating estimates of relative risk into absolute risk for a meaningful time period |  |  |

Continued on next page

| Other analyses | 17 | Report other analyses done—eg analyses of subgroups and interactions, and sensitivity analyses |  |  |
| --- | --- | --- | --- | --- |
| Discussion | | | | |
| Key results | 18 | Summarise key results with reference to study objectives | 20 | Variation of Mean level of vitamin D |
| Limitations | 19 | Discuss limitations of the study, taking into account sources of potential bias or imprecision. Discuss both direction and magnitude of any potential bias | 19 | This research aimed to find how vitamin D level correlates with SLEDAI. No relationship association between SLEDAI and the level of vitamin D is observed using ML. T |
| Interpretation | 20 | Give a cautious overall interpretation of results considering objectives, limitations, multiplicity of analyses, results from similar studies, and other relevant evidence | 22 | The study observed a high prevalence of vitamin D insufficiency, particularly among females, suggesting that vitamin D may play a crucial role in SLE patients, who are mostly female. The majority of SLE patients in this study had low vitamin D levels and high SLEDAI scores. |
| Generalisability | 21 | Discuss the generalisability (external validity) of the study results | 19 | It is observed that there is a positive relationship between age and vitamin D, |
| Other information | |  | | |
| Funding | 22 | Give the source of funding and the role of the funders for the present study and, if applicable, for the original study on which the present article is based | 22 | This study does not take into account corticosteroid therapy, complement levels, anti-dsDNA antibody titers, concomitant rheumatologic therapies, and ongoing vitamin D or calcium supplementation. However, future research will be conducted to include these factors. |

*Give information separately for cases and controls in case-control studies and, if applicable, for exposed and unexposed groups in cohort and cross-sectional studies.

**Note:** An Explanation and Elaboration article discusses each checklist item and gives methodological background and published examples of transparent reporting. The STROBE checklist is best used in conjunction with this article (freely available on the Web sites of PLOS Medicine at http://www.plosmedicine.org/, Annals of Internal Medicine at http://www.annals.org/, and Epidemiology at http://www.epidem.com/). Information on the STROBE Initiative is available at www.strobe-statement.org.
