# Supplementary material for: Leveraging machine learning to evaluate factors influencing vitamin D insufficiency in SLE patients: A case study from southern Bangladesh
Source: PLOS Glob Public Health. 2023 Oct 31;3(10):e0002475. doi: 10.1371/journal.pgph.0002475 (PMC10617712; doi:10.1371/journal.pgph.0002475)
Supplement: S2 File — (DOCX) [file pgph.0002475.s002.docx]

**
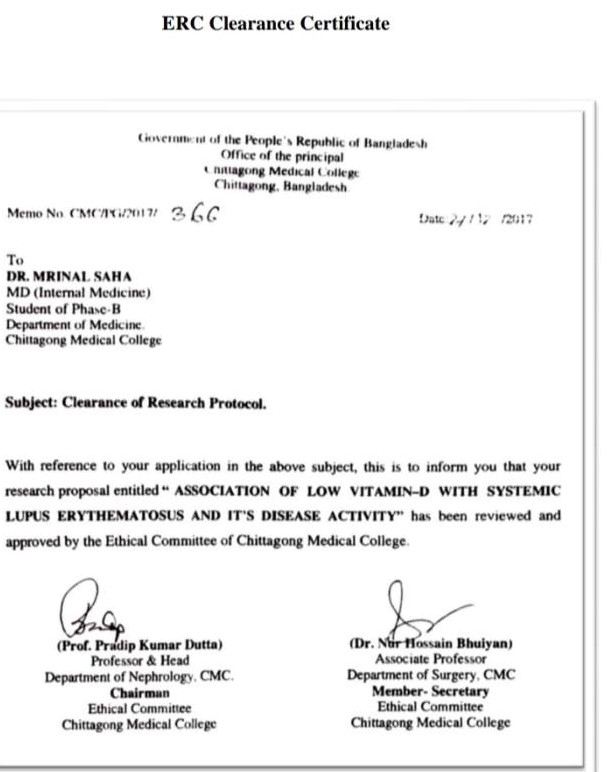
**

**Case Record Form**

**TITLE : Association of low Vitamin-D with Systemic Lupus**

**Erythematosus and it’s disease activity.**

Subject : ________________ Visit Date: ____/____/________

**Part-1**

**Demographics and Socioeconomic Data at Diagnosis**

Hospital Registration No: _____________

Name : _________________________________ Age : ______________ in years

Address: ________________________________________________________

________________________________________________________________

________________________________________________________________

Mobile No: ___________________

Sex : 1- Male 2- Female

Marital status : 1- Single 2- Married 3 – Widow 4 - Separated/Divorced

Education: 0 - No Education 1 – Primary 2 - Secondary

3 - Higher Secondary 4 - Graduate

5 - Others (Specify) _____________

Ethnicity : 1 – Non-tribes 2 – Tribes

Religion : : 1 - Islam 2 – Hindu 3- Buddhist 4 – Christian 5 – Others

Occupation: 1 - Self-employed 2 - Professional 3- Official employee

4 - House wife 5 - Student/Dependent 6 - Unemployed

7 - Retired 8 - Others ________________

Monthly household income (in Taka) : _____________________

Height: __________ cm Weight: _________ kg BMI: _________

**Personal History**

Use of Contraceptive : 1 - Yes 2 - No 3 - Not Applicable

If yes please Specify duration: _____________ in years

Use of Contraceptive :1 - OCP 2 – Injectable contraceptive

3 – IUCD4 – Implant

Menstrual history (Only for woman):

1 - Menstrual Cycle- 1-regular 2-Irregular

2 - Delivery 1-Normal 2- Abnormal

3 - Abortion- 1-Yes 2-No

4 - Toxemias – 1- Yes 2-No

Family History of CTD : 1 - Yes 2 - No 3 - Not Applicable

If yes specify:____________

Immediate of 1^st^degree :*(CTD/relation)*: 1. Parents 2. Siblings 3. Off-spring

2^nd^ or 3^rd^degre: *(CTD/relation/Maternal or paternal)* (please specify): _________________

Exposure to Sunlight : ________hr/Day

Use of sun protectors : Sunscreen umbrella others ______

Clothings : Veil Hijab Borkha Others________

Year SLE Diagnosed: ____________________________Examination Date : ______________

**Revised American Rheumatism Association (ACR) Criteria-for SLE Diagnosis**

| **Characteristics** | **Ever**  **Involved** | **Currently**  **Involved** | **Comments** |
| --- | --- | --- | --- |
| **Malar Rash** |  Yes  No  Year ______ |  Yes  No  ______ | Fixed erythema, flat or raised, sparing thenasolabial folds. |
| **Discoid rash** |  Yes  No  Year ______ |  Yes  No  ______ | Erythematous raised patches with adherent  keratotic scarring and follicular plugging |
| **Photosensitivity** |  Yes  No  Year ______ |  Yes  No  ______ | Rash due to unusual reaction to sunlight |
| **Oral ulcers** |  Yes  No  Year ______ |  Yes  No  ______ | Oral or nasopharyngeal ulceration, which  may be painless |
| **Arthritis** |  Yes  No  Year ______ |  Yes  No  ______ | Non-erosive, involving two or more  peripheral joints |
| **Serositis** |  Yes  No  Year ______ |  Yes  No  ______ | Pleuritis (history of pleuritic pain or rub, or  pleural effusion) *or* pericarditis (rub, ECG  evidence or effusion) |
| **Renal disorder** |  Yes  No  Year ______ |  Yes  No  ______ | Persistent proteinuria >0.5 g/day  *Or*cellular casts (red cell, granular or tubular) |
| **Neurological disorder** |  Yes  No  Year ______ |  Yes  No  ______ | Seizures or psychosis, in the absence of  provoking drugs or metabolic derangement |
| **Haematological disorder** |  Yes  No  Year ______ |  Yes  No  ______ | Haemolyticanaemia (Coomb’s positive)  *or* leucopenia* (<4 ×10^9^/L)  *or* lymphopenia* (<1 × 10^9^/L)  *or*thrombocytopenia* (<100 × 10^9^/L) in theabsence of offending drugs |
| **Immunological disorder** |  Yes  No  Year ______ |  Yes  No  ______ | Anti-DNA antibodies in abnormal titre  *Or*presence of antibody to Sm antigen  *Or*positive antiphospholipid antibodies |
| **ANA disorder** |  Yes  No  Year ______ |  Yes  No  ______ | Abnormal titre of ANA byimmunofluorescence. |
| **A person has SLE if any 4 out of these 11 features are presentserially or simultaneously.** | | | |
| ***On two separate occasions.**  If any of the above are currently involved, add current treatments to prior and comment section.  Comments: _______________________________________________________________________ | | | |

**2015 ACR/SLICC revised Criteria for Diagnosis of SLE**

| **Criteria** | **Score** | | **Comment** |
| --- | --- | --- | --- |
| **1. Acute / Sub Acute Cutaneous lupus rash :** | **Upto 2 points** | |  |
| - Malar Rash |  Yes  No | **2** |  |
| - Subacute cutaneous Lupus Erythematosus(SCLE) rash |  Yes  No | **1** |  |
| - Palpable purpura or urticarial Vasculitis |  Yes  No | **1** |  |
| - Photosensitivity |  Yes  No | **1** |  |
| **2. Discoid lupus erythometosus (DLE) rash or hypertrophic Lupus rash** |  Yes  No | **1** |  |
| **3. Non-scarring frank alopesia** |  Yes  No | **1** |  |
| **4. Oral /Nasal ulcers** |  Yes  No | **1** |  |
| **5. Joint disease** |  Yes  No | **1** |  |
| **6. Pleurisy and /or Pericarditis** |  Yes  No | **1** |  |
| **7. Neurological : Psychosis and /or seizure and / or acute confusion** |  Yes  No | **1** |  |
| **8. Kidney involvement :** | **Upto 2 points** | |  |
| - Proteinuria ≥ 3^+^ or ≥ 500 mg/ day or urinary casts |  Yes  No | **1** |  |
| - Biopsy – proven nephritis compatible with SLE |  Yes  No | **2** |  |
| **9. Hematologic :** | **Upto3 points** | |  |
| - WBC Count < 4000 /mm^3^or lymphocyte count < 1500 mm^3^   On 2 occasions or WBC count < 4000 /mm^3^along with lymphocyte count < 1500 mm^3^ in one occasion . |  Yes  No | **1** |  |
| - Thrombocytopenia < 100,000/ mm^3^ |  Yes  No | **1** |  |
| - Hemolytic Anemia ( Coomb’s Test Positive) |  Yes  No | **1** |  |
| **10. Serological Test** | **Upto3 points** | |  |
| - Low titre positive ANA |  Yes  No | **1** |  |
| - High titer FANA with homogenous or rim pattern |  Yes  No | **2** |  |
| - Positive anti-ds DNA |  Yes  No | **2** |  |
| - Positive anti-Sm |  Yes  No | **2** |  |
| - Anti- Phospholipid antibodies (aPLs) |  Yes  No | **1** |  |
| - Low serum Complement ( C_3_ and /or C_4_ / or CH_50_) |  Yes  No | **1** |  |

**SLICC (Systemic Lupus International Collaborating Clinics ) Criteria : Total Score : _________**

The patients with 4 points out of 16, have definite diagnosis of SLE. With 3 points highly suggestive SLE, with 2 points probable SLE and with one point possible SLE are the diagnosis.

**Renal History**  Yes  No

Year of 1^st^ instance of significant proteinuria (500 mg/day) :__________________________

Renal Biopsy Date and Report: ________________________________________________

Lupus Nephritis Type : _______________________________________________________

Year Lupus Nephritis diagnosed _______________

Any Previous Dialysis? Yes No

If “Yes”, provide Start date of Last Dialysis ____/____/_______

DD MM YYYY

If “Yes”, provide Start date of Last Dialysis ____/____/_______

DD MM YYYY

**Treatment Received**

| Name of Treatment | Given | Dose | Unit | Frequency | Route | Start Date | End Date |
| --- | --- | --- | --- | --- | --- | --- | --- |
| NSAIDS |  Yes  No |  |  |  |  |  |  |
| Prenisolone |  Yes  No |  |  |  |  |  |  |
| Azathioprine |  Yes  No |  |  |  |  |  |  |
| HydroxyChloroquine |  Yes  No |  |  |  |  |  |  |
| Cyclophosphamide |  Yes  No |  |  |  |  |  |  |
| Other: |  Yes  No |  |  |  |  |  |  |
| Other: |  Yes  No |  |  |  |  |  |  |
| Other: |  Yes  No |  |  |  |  |  |  |
| Vitamin D ( Previous history – More than 2 months before ) | | | | | | | |

**Vitamin D Levels :**

1. Date : Value : Diagnostic Centre :

2. Date : Value : Diagnostic Centre :

**Laboratory tests of SLE Patients**

| **Name of the Diagnosis** | **Diagnosis done** | **Value** | **Unite** | **Date** |
| --- | --- | --- | --- | --- |
| Hemoglobin |  Yes  No |  | g/l |  |
| ESR |  Yes  No |  | mm in 1^st^hr |  |
| RBC |  Yes  No |  |  |  |
| WBC |  Yes  No |  | 10^9^/L |  |
| Platelet |  Yes  No |  | 10^9^/L |  |
| CRP |  Yes  No |  |  |  |
| ANA |  Yes  No |  |  |  |
| Anti DsDNA |  Yes  No |  |  |  |
| C3 |  Yes  No |  | g/L |  |
| C4 |  Yes  No |  | g/L |  |
| S.Electrolytes |  Yes  No |  |  |  |
| S.Creatinine |  Yes  No |  | μmol/L |  |
| RBS |  Yes  No |  | mmol/L |  |
| S. Albumin |  Yes  No |  | g/L |  |
| HBsAg |  Yes  No |  |  |  |
| Anti HBC |  Yes  No |  |  |  |
| Prothrombin time (PT) |  Yes  No |  |  |  |
| Urine-Albumin |  Yes  No |  |  |  |
| Urine-WBC |  Yes  No |  |  |  |
| Urine-RBC |  Yes  No |  |  |  |
| Urine-Casts |  Yes  No |  |  |  |
| Serum Urea |  Yes  No |  | μmol/L |  |
| S.TSH |  Yes  No |  |  |  |
| S.Ca^+2^ |  Yes  No |  |  |  |
| 24 hr urinary total protein |  Yes  No |  |  |  |
| Renal Biopsy |  Yes  No |  |  |  |
| X-Ray Chest |  Yes  No |  |  |  |
| USG of W/A |  Yes  No |  |  |  |
| Echocardiography |  Yes  No |  |  |  |
| Other: |  |  |  |  |
|  |  Yes  No |  |  |  |
|  |  Yes  No |  |  |  |
|  |  Yes  No |  |  |  |

| **Fatigue Severity Scale Questionnaire**  **Instructions: Circle the number that best represents your response to each question.**  **Scoring range: 1=strongly disagree with the statement**  **7=strongly agree with the statement.**   \| **During the past week , I have found that :** \| \| **Score** \| \| \| \| \| \| \| \| --- \| --- \| --- \| --- \| --- \| --- \| --- \| --- \| --- \| \|  \| **Complaints** \|  \|  \|  \|  \|  \|  \|  \| \| 1 \| My motivation is lower when I am fatigue. \| 1 \| 2 \| 3 \| 4 \| 5 \| 6 \| 7 \| \| 2 \| Exercise brings on my fatigue . \| 1 \| 2 \| 3 \| 4 \| 5 \| 6 \| 7 \| \| 3 \| I am easily fatigued . \| 1 \| 2 \| 3 \| 4 \| 5 \| 6 \| 7 \| \| 4 \| Fatigue interferes with my physical functioning. \| 1 \| 2 \| 3 \| 4 \| 5 \| 6 \| 7 \| \| 5 \| Fatigue causes frequent problems for me. \| 1 \| 2 \| 3 \| 4 \| 5 \| 6 \| 7 \| \| 6 \| My fatigue prevents sustained physical functioning. \| 1 \| 2 \| 3 \| 4 \| 5 \| 6 \| 7 \| \| 7 \| Fatigue interferes with carrying out certain duties and responsibilities . \| 1 \| 2 \| 3 \| 4 \| 5 \| 6 \| 7 \| \| 8 \| Fatigue is among my three most disabling symptoms. \| 1 \| 2 \| 3 \| 4 \| 5 \| 6 \| 7 \| \| 9 \| Fatigue interferes with my work , family or social life . \| 1 \| 2 \| 3 \| 4 \| 5 \| 6 \| 7 \| |
| --- | --- | --- | --- | --- | --- | --- | --- | --- | --- | --- | --- | --- | --- | --- | --- | --- | --- | --- | --- | --- | --- | --- | --- | --- | --- | --- | --- | --- | --- | --- | --- | --- | --- | --- | --- | --- | --- | --- | --- | --- | --- | --- | --- | --- | --- | --- | --- | --- | --- | --- | --- | --- | --- | --- | --- | --- | --- | --- | --- | --- | --- | --- | --- | --- | --- | --- | --- | --- | --- | --- | --- | --- | --- | --- | --- | --- | --- | --- | --- | --- | --- | --- | --- | --- | --- | --- | --- | --- | --- | --- | --- | --- | --- | --- | --- | --- | --- | --- | --- |

**FSS Scoring : Add up the circled numbers and divide by 9 = __________**

**Compare Result with the following scores :**

People who do not experience fatigue score about 2.8

People with Lupus score about 4.6

People with lime disease score about 4.8

People with fatigue Related to Multiple Sclerosis scores about 5.1

People with Chronic fatigue syndrome scores about 6.1

**SLEDAI**

| **Descriptor** | **Points** | **Present or in last 10 days** | **Not Done** |
| --- | --- | --- | --- |
| Seizure | 8 | Yes No |  |
| Psychosis | 8 | Yes No |  |
| Organic brain syndrome | 8 | Yes No |  |
| Visual Disturbance | 8 | Yes No |  |
| Cranial nerve disorder | 8 | Yes No |  |
| Lupus headache | 8 | Yes No |  |
| Corobrovascular accident (CVA) | 8 | Yes No |  |
| Vasculitis | 8 | Yes No |  |
| Arthritis | 4 | Yes No |  |
| Mysositis | 4 | Yes No |  |
| Urinary casts | 4 | Yes No |  |
| Hematuria | 4 | Yes No |  |
| Proteinuria | 4 | Yes No |  |
| Pyuria | 4 | Yes No |  |
| New rash | 2 | Yes No |  |
| Alopecia | 2 | Yes No |  |
| Mucosal ulcers | 2 | Yes No |  |
| Pleurisy | 2 | Yes No |  |
| Pericarditis | 2 | Yes No |  |
| Low complement | 2 | Yes No |  |
| Increase DNA binding | 2 | Yes No |  |
| Fever | 1 | Yes No |  |
| Thrombocytopenia | 1 | Yes No |  |
| Leukopenia | 1 | Yes No |  |
| **Total Score** |  |  |  |

| □ Mild or Moderate | □ Flare Severe Flare |
| --- | --- |
| □ Change in SLEDAI > 3 points | □ Change in SLEDAI > 12 |

**Comments:________________________________________________________________**

| Retinal change or optic atrophy?  SLICC/ACR Damage Index | Yes, Single episode | Yes, repeat episode | No |
| --- | --- | --- | --- |
| Retinal change: Documented by ophthalmologic examination, may result in field defect, legal blindness |  |  |  |
| Optic atrophy: Documented by ophthalmologic examination |  |  |  |
| Cataract? | Yes | No |  |
| A lens opacity in either eye, ever, whether primary or secondary to steroid therapy, documented by ophthalmoscopy |  |  |  |
| Cognitive Impairment or Major Psychosis?  Cognitive impairment: Memory deficit, difficulty with calculation, poor concentration, difficulty in spoken or written language, impaired performance level, documented on clinical examination or by formal neurocognitive testing.  Major psychosis: Altered ability to function in normal activity due to psychiatric reasons. Severe disturbance in the perception of reality characterized by the following features: delusions, hallucinations, incoherence, marked loose associations, impoverished thought content, marked illogical thinking, bizarre, disorganized or catatonic behavior. | Yes | No |  |
| Seizures requiring therapy for >6 months? | Yes | No |  |
| Cerebrovascular Accident?  Also includes surgical resection for causes other than malignancy. | Single episode | 2 or more episode | No |
| Cranial for Peripheral Neuropathy?  Damage to either a cranial or peripheral nerve (excluding optic) resulting in either motor or sensory dysfunction. | Yes | No |  |
| Transverse Myelitis?  Lower extremity weakness or sensory loss with loss of rectal and urinary bladder sphincter cotnrol. | Yes | No |  |
| Estimated or Measured GFR <50%? | Yes | No |  |
| Proteinuria >3.5 g/24 hours? | Yes | No |  |
| End-stage renal disease? | Yes | No |  |
| Pulmonary Hypertension? | Yes | No |  |
| Pulmonary Fibrosis? | Yes | No |  |
| Shrinking Lung? | Yes | No |  |
| Pleural Fibrosis? | Yes | No |  |
| Pulmonary Infarction? | Yes | No |  |
| Angina or Coronary Artery Bypass? | Yes | No |  |
| Myocardial Infarction? | Single episode | More than 1 episode | No |
| Cardiomyopathy? | Yes | No |  |
| Valvular disease? | Yes | No |  |
| Pericarditis or Pericardiectomy? | Yes | No |  |
| Claudication?  Persistent for 6 months by history | Yes | No |  |
| Minor Tissue Loss form Peripheral Vascular Disease?  Such as pulp space | Yes | No |  |
|  |  |  |  |
| Significant Tissue Loss from Peripheral Vascular Disease?  At least loss or resection of a digit | Single episode | 2 or more episodes | No |
| Stricture or Upper Gastrointestinal Tract Surgery? | Yes | No |  |
| Pancreatic Insufficiency Requiring Enzyme Replacement or with Pseudocyst? | Yes | No |  |
| Muscle Atrophy or Weakness?  Demonstrated on clinical exam | Yes | No |  |
| Deforming or Erosive Arthritis?  Including reducible deformities, excluding avascular necrosis | Yes | No |  |
| Venous Thrombosis with Swelling, Ulceration or Venous Stasis? | Yes | No |  |
| Infarction or Resection of Bowel (below duodenum), Spleen, Liver or Gallbladder? | 1 site | 2 or more sites | No |
| Mesenteric Insufficiency? | Yes | No |  |
| Chronic Peritonitis? | Yes | No |  |
| Osteoporosis with Fracture or Vertebral Collapse?  Excluding AVN | Yes | No |  |
| Avascular Necrosis?  Demonstrated on imaging | Single episode | 2 or more episodes | No |
| Scarring Chronic Alopecia? | Yes | No |  |
| Extensive Scarring or Panniculum other than Scalp and Pulp Space? | Yes | No |  |
| Skin Ulceration (excluding thrombosis)? | Single site | 2 or more sites | No |
| Premature Gonadal Failure?  Including secondary amenorrhea | Yes | No |  |
| Diabetes Requiring Therapy? | Yes | No |  |
| Malignancy?  Documented by pathology, excluding dysplasia | Single site | 2 or more sites |  |

**CONSENT FORM FOR PARTICIPATION IN RESEARCH**

I……………………………………………………………………...........being over the age of 18 years hereby consent to participate in the research project,

**“Vitamin-D level in Systemic Lupus Erythematosus patients and its association with disease activity”**

1. Have read the information provided.

2. Details of procedures and any risks have been explained to my satisfaction.

3. I agree to audio/video recording of my patients’ information and participation.

4. I am aware that I should retain a copy of the information Sheet and Consent Form for future reference

5. I understand that:

- I or my patient may not directly benefit from taking part in this research.

- I am free to withdraw from the project at anytime and am free to decline to answer particular questions while the information gained in this study will be published as explained.

- I will not be identified, and individual information will remain confidential.

- Whether I participate or not, or withdraw after participating, will have no effect on any treatment or service that is being provided to me

- I may ask that the recording/observation be stopped at anytime, and that I may withdraw at anytime from the session or the research without disadvantage

- I agree/do not agree to the transcript being made available to other researchers who are not members of this research team, but who are judged by the research team to be doing related research, on condition that my identity is not revealed.

I have had the opportunity to discuss taking part in this research with a family member or friend

**Participant’s**

**signature………………………………………Date…………………………………….**

**.**

I certify that I have explained the study to the volunteer and consider that she/he understands what is involved and freely consents to participation.

**Researcher’s name………………………………………………………………………..**
