# Supplementary material for: Leveraging machine learning to evaluate factors influencing vitamin D insufficiency in SLE patients: A case study from southern Bangladesh
Source: PLOS Glob Public Health. 2023 Oct 31;3(10):e0002475. doi: 10.1371/journal.pgph.0002475 (PMC10617712; doi:10.1371/journal.pgph.0002475)
Supplement: Attachment — Submitted filename: Response to Reviewers comments.docx [file pgph.0002475.s003.docx]

**Journal Requirements:**

1. Please send a completed 'Competing Interests' statement, including any COIs declared by your co-authors. If you have no competing interests to declare, please state "The authors have declared that no competing interests exist". Otherwise please declare all competing interests beginning with the statement "I have read the journal's policy and the authors of this manuscript have the following competing interests:"

Answer: Thank you. We added this statement at the end of the manuscript.

2. We have noticed that you have uploaded Supporting Information files, but you have not included a list of legends. Please add a full list of legends for your Supporting Information files after the references list.

Answer: Thank you. We added a legend after the references list.

3. Please provide separate figure files in .tif or .eps format only and remove any figures embedded in your manuscript file. Please also ensure all files are under our size limit of 10MB.

For more information about figure files please see our guidelines:

https://journals.plos.org/globalpublichealth/s/figures

https://journals.plos.org/globalpublichealth/s/figures#loc-file-requirement

Answer: We removed all the figures from the manuscript and we added a separate figure file. We converted all the figures in .tif as well.

4. Fig 1: please (a) provide a direct link to the base layer of the map (i.e., the country or region border shape) and ensure this is also included in the figure legend; and (b) provide a link to the terms of use / license information for the base layer image or shapefile. We cannot publish proprietary or copyrighted maps (e.g. Google Maps, Mapquest) and the terms of use for your map base layer must be compatible with our CC-BY 4.0 license.

Note: if you created the map in a software program like R or ArcGIS, please locate and indicate the source of the basemap shapefile onto which data has been plotted.

If your map was obtained from a copyrighted source please amend the figure so that the base map used is from an openly available source. Alternatively, please provide explicit written permission from the copyright holder granting you the right to publish the material under our CC-BY 4.0 license.

Please note that the following CC BY licenses are compatible with PLOS license: CC BY 4.0, CC BY 2.0 and CC BY 3.0, meanwhile such licenses as CC BY-ND 3.0 and others are not compatible due to additional restrictions.

If you are unsure whether you can use a map or not, please do reach out and we will be able to help you. The following websites are good examples of where you can source open access or public domain maps:

* U.S. Geological Survey (USGS) - All maps are in the public domain. (http://www.usgs.gov)

* PlaniGlobe - All maps are published under a Creative Commons license so please cite “PlaniGlobe, http://www.planiglobe.com, CC BY 2.0” in the image credit after the caption. (http://www.planiglobe.com/?lang=enl)

* Natural Earth - All maps are public domain. (http://www.naturalearthdata.com/about/terms-of-use/)

Answer: We updated Figure 1 according to the guidelines. We created it ourselves. Base layer image is incorporated in the figure.

Reviewers' comments:

1. Does this manuscript meet PLOS Global Public Health’s publication criteria? Is the manuscript technically sound, and do the data support the conclusions? The manuscript must describe methodologically and ethically rigorous research with conclusions that are appropriately drawn based on the data presented.

Reviewer #1: Yes

Reviewer #2: Yes

Answer: Thank you very much.

2. Has the statistical analysis been performed appropriately and rigorously?

Reviewer #1: Yes

Reviewer #2: Yes

Answer: Thank you very much.

3. Have the authors made all data underlying the findings in their manuscript fully available (please refer to the Data Availability Statement at the start of the manuscript PDF file)?

The PLOS Data policy requires authors to make all data underlying the findings described in their manuscript fully available without restriction, with rare exception. The data should be provided as part of the manuscript or its supporting information, or deposited to a public repository. For example, in addition to summary statistics, the data points behind means, medians and variance measures should be available. If there are restrictions on publicly sharing data—e.g. participant privacy or use of data from a third party—those must be specified.

Reviewer #1: No

Reviewer #2: Yes

Answer: Thank you very much. We made all data available in the manuscript during the submission process. Here is the link again: <https://docs.google.com/spreadsheets/d/1XKlP4MwJKb15SNg-k1x96y5_NiBGcQlU7o-P7oostvw/edit?usp=sharing>

4. Is the manuscript presented in an intelligible fashion and written in Standard English?

PLOS Global Public Health does not copyedit accepted manuscripts, so the language in submitted articles must be clear, correct, and unambiguous. Any typographical or grammatical errors should be corrected at revision, so please note any specific errors here.

Reviewer #1: Yes

Reviewer #2: Yes

Answer: Thank you very much.

Reviewer #1: Dear author(s), your statistical analysis part is good with neat explanations. But coming to ML approaches, the RF approach is susceptible to overfitting, so my suggestion is to try other models and make a fair comparison with those approaches. Do feature selections properly. If possible give a sample dataset in the article. Also, the accuracies of models should be noted and compared. You can also try a deep model like CNN to justify whether your ML models are suitable for this type of application over DL models or not.

Answer:

Thank you for your valuable suggestions and feedback. We appreciate your concern regarding the possibility of overfitting in our Random Forest (RF) model and agree with the importance of rigorous model selection and validation practices. To address overfitting, we have implemented a 3-Fold cross-validation strategy to optimize the RF model's hyperparameters (max_depth = 2, n_estimators = 50). Cross-validation provides a robust estimate of the model's performance on unseen data, mitigating the risk of overfitting (James et. Al., 2013). Additionally, we have applied bootstrapping to generate confidence intervals, further strengthening our models by providing uncertainty estimates. We have compared the RF model's performance to a simpler Linear Regression (LR) model, as you suggested. Given the small number of features (p=10) relative to observations (n=50) in our data, we did not find feature selection necessary (James et. Al., 2013). Instead, we have provided an in-depth analysis of feature importance through RF feature importance, Permutation-based feature importance, and SHAP values. Our task is regression, for which we have used RMSE and MAE, commonly used measures for such tasks.

We appreciate your suggestion to explore Convolutional Neural Networks (CNN). However, our dataset, being small and lacking the unstructured grid-like topology like images or text, may not benefit from such complex models. Deep learning models like CNN generally require larger unstructured grid-like topology datasets and risk overfitting when applied to small datasets (Goodfellow et al., 2016). Moreover, the principle of Occam's Razor (Bargagli et al. 2022) suggests a preference for simpler models when they perform adequately. This principle is relevant to our situation, given the trade-off between model complexity, interpretability, and the risk of overfitting. As the complexity or flexibility of a model increases, its interpretability often decreases, and the risk of overfitting rises (Fig. 1) .

**Fig 1:** A representation of the tradeoff between flexibility and interpretability, using different statistical learning methods.

Complex models with many parameters, such as deep learning models, can overfit small datasets by learning not just the underlying patterns but also the random noise. Conversely, simpler models like LR or RF are often more interpretable and less prone to overfitting, particularly when the amount of training data is limited (Hastie et al., 2009)). Therefore, for our study, focusing on inference rather than prediction, more interpretable models are preferable.

**References:**

Goodfellow, I., Bengio, Y., & Courville, A. (2016). Deep Learning. MIT Press.

James, G. and Witten, D. and Hastie, T. and Tibshirani, R. (2014). An Introduction to Statistical Learning: with Applications in R. (Springer Series in Statistics).

Hastie, Trevor & Tibshirani, Robert & Friedman, Jerome. (2009). The Elements of Statistical Learning: Data Mining, Inference, and Prediction, Second Edition (Springer Series in Statistics).

Bargagli Stoffi, F.J., Cevolani, G. & Gnecco, G. Simple Models in Complex Worlds: Occam’s Razor and Statistical Learning Theory. Minds & Machines 32, 13–42 (2022).

Reviewer #2:

1. The title is unclear.

Answer: Thank you for your feedback on the title. We aimed to convey the core aspects of our research, but we understand that clarity is crucial. We revised it to improve its clarity and ensure it accurately reflects the content of our paper. The revised title is:

"**Leveraging Machine Learning to Evaluate Factors Influencing Vitamin D Insufficiency in SLE Patients: A Case Study from Southern Bangladesh**". We believe this title better encapsulates the geographical focus, the patient group under study, and the machine learning methods we utilized. Please let us know if you have any suggestions.

2. The results section of the abstract of the article does not report any results or values quantitatively.

Answer: Thank you for the suggestion. We have incorporated quantitative scores into the abstract for added clarity and precision.

3. The materials and methods section of the abstract is written very unintelligibly.

Answer: Thank you for your feedback. We understand that the Methods' section of the abstract was not clear. We have taken your comments into consideration and made revisions to improve its clarity and intelligibility. We believe these changes will help readers better understand our methods and approach.

4. The motivation is not clear. Why was this work done? What problem does it address that previous methods could not?

Answer: Thank you for your insightful comment. This research addresses two prominent issues. The primary motivation is the high prevalence of vitamin D insufficiency in SLE patients, especially in the southern part of Bangladesh. Understanding the contributing factors to this condition and its potential impact on disease activity is crucial for patient care. Previous studies primarily utilized conventional statistical methods such as correlation-based analysis and t-tests, which often overlooked potential nonlinear relationships between variables or the influence of multiple variables on vitamin D levels simultaneously. These methods have limitations in capturing complex relationships, which can leave significant patterns unidentified. To address these gaps, this study employs advanced machine learning techniques, specifically Linear Regression and Random Forest. These methodologies are capable of capturing intricate patterns and relationships, including nonlinear dependencies and interactions among variables. They can also rank the importance of different features, yielding more insights into the key factors affecting vitamin D levels. By integrating these machine learning models with traditional statistical analyses, our research aims to provide a comprehensive understanding of the dynamics influencing vitamin D levels in SLE patients. This innovative approach addresses the limitations of previous methods and enhances our understanding of vitamin D insufficiency in SLE patients. The findings from this study can potentially contribute to better clinical management strategies for SLE patients and drive further investigations in this field.

5. Some state-of-the-art papers on CNN and machine learning in the field of paper should be taken into account, such as:

(https://link.springer.com/chapter/10.1007/978-3-030-98253-9_20)

(https://bmcbioinformatics.biomedcentral.com/articles/10.1186/s12859-022-04965-8)

(https://ro-journal.biomedcentral.com/articles/10.1186/s13014-021-01906-2)

(https://link.springer.com/chapter/10.1007/978-3-031-27420-6_7)

Answer: We are really sorry. None of these references are connected to our research. So, we did not include them in the manuscript. More detailed explanations are given in our response one.

6. Add a Related Work section to the paper.

Answer: Thank you. Added to the manuscript.

7. Include a comparison table in the related work that highlights the strengths and weaknesses of the proposed method as well as previous methods.

Answer:

Thank you for your insightful suggestion. Here is a comparison table that highlights the strengths and weaknesses of our method compared to previous methods:

| Methods | Strengths | Weaknesses |
| --- | --- | --- |
| Correlation and t-tests (Previous Methods) | Simple to understand and interpret, Widely accepted in the scientific community | May not detect complex, nonlinear relationships, Cannot handle high-dimensional data well, Cannot easily provide insights into the relative importance of features |
| Linear Regression (Our Method) | Can model linear relationships, Provides coefficients that can be interpreted as the strength of the relationship between each feature and the outcome | Assumes a linear relationship, Can be sensitive to outliers |
| Random Forest (Our Method) | Can model nonlinear and complex relationships, Robust to outliers, Provides intrinsic feature importance | Requires careful tuning to avoid overfitting, Interpretability can be more challenging than linear methods |

The table above illustrates that while traditional methods like correlation and t-tests are simple and widely accepted, they might not capture complex relationships in the data. On the other hand, our proposed methods, Linear Regression and Random Forest, are capable of modeling such complex relationships and providing insights into the relative importance of features. However, they also come with their own challenges, such as the need for careful tuning and the interpretability of the results. We believe that the combined use of traditional statistical tests and machine learning models in our study provides a more comprehensive understanding of the factors influencing vitamin D levels in SLE patients.

8. The novelty of the algorithm needs to be incorporated.

Answer: Thank you for your valuable comment. In this study, while we utilize established algorithms, Linear Regression and Random Forest, the novelty lies in their application to the specific problem of vitamin D insufficiency in SLE patients and the combination of these algorithms with an extensive feature importance analysis.

Traditionally, studies investigating vitamin D insufficiency in SLE patients have relied on conventional statistical methods. However, our approach leverages the power of machine learning to uncover potentially intricate patterns and relationships in the data that could not be detected by standard techniques.

For instance, we performed an in-depth feature importance analysis using the Random Forest's intrinsic feature importance, permutation-based feature importance, and SHAP values, which helps to understand the relative contribution of each feature to the model's predictions. This is a significant advancement over traditional statistical methods and can provide much richer insights.

Moreover, the integration of these machine learning models with classical statistical methods, such as Student's t-tests and correlation analyses, adds a further layer of novelty to our approach.

In summary, while the algorithms themselves are well-established, the novelty of our work lies in the innovative application and combination of these methods to provide a more comprehensive understanding of vitamin D insufficiency in SLE patients. This approach could open new avenues for the application of machine learning in medical research and patient care.

9. Describe how to deal with overfitting in your model.

Answer: Thank you for your question. In this study, overfitting was mitigated through several strategies:

Cross-validation: A 3-Fold cross-validation strategy was employed, which helps to ensure that the model generalizes well to unseen data. In cross-validation, the dataset is divided into 'k' subsets or 'folds'. The model is then trained on k-1 folds and tested on the remaining fold. This process is repeated k times, with a different fold used as the testing set each time. This approach provides a robust estimate of the model's predictive performance.

Hyperparameter tuning: Optimal values for the model's hyperparameters were selected through a grid search approach, which finds the combination of hyperparameters that result in the best cross-validation performance.

Feature importance analysis: Through feature importance analysis methods like permutation feature importance and SHAP values, irrelevant features, or 'noise', were identified. Models can overfit if they learn patterns from noise, so this analysis helped to ensure that the models focused on the genuine, informative features.

Ensemble method: The Random Forest algorithm was used, which is an ensemble of decision trees. By averaging the predictions of multiple decision trees, the Random Forest model effectively reduces the risk of overfitting.

Lastly, we validated our approach by computing bootstrapped confidence intervals. This provided robust uncertainty estimates that further ensured the prevention of overfitting.

These steps helped to ensure that our model had good predictive performance, not just on the training data, but also on unseen data.

10. Besides the language, the paper needs improvement.

Answer: The manuscript is updated as suggested.
